# Pyorrhœa Alveolaris

**Published:** 1899-07

**Authors:** Theo. V. Smith

**Affiliations:** University of Pennsylvania


### PYORRHŒA ALVEOLARIS.

BY THEO. V. SMITH, UNIVERSITY OF PENNSYLVANIA.

MRS. R., of Philadelphia. This case came into my hands through the clinic on April 4, 1899. The teeth affected were eight lower anterior (two supernumerary lateral incisors), two upper central incisors, and two upper molars, second and third on each side. The lower teeth were very loose, particularly the lateral incisors, and covered with a salivary deposit. No history of gouty troubles or rheumatism could be obtained. The upper teeth were not so much affected, and readily responded to treatment. The teeth were as thoroughly cleansed as possible near the apex of root, and polished with pumice and chalk. The gums and sockets were then well soaked and washed clean with pyrozone, about three per cent., and an application of sulphuric acid, twenty per cent., made to produce a granulating surface, and the pockets packed with sulphate of quinine. The teeth were also ligatured to allow surgical rest. The mouth gave indications of an acid reaction, and the patient was instructed to spread prepared chalk over the teeth at night, and use a wash composed of hydronaphthol, fifteen grains, alcohol and aquæ destillatæ, one ounce, about a teaspoonful in a half-glass of water, three times a day.

Marked improvement was noted, and the patient very much encouraged and gratified. After several applications of sulphate of quinine the gum tissue seemed to be in an almost normal condition, with the exception of tightening around the teeth. No pus or exudate could be seen. Sulphate of quinine is now occasionally applied, and the mouth-wash used as before, to keep up an aseptic condition and encourage tightening.

The patient from the first marked a degree of comfort which had not been enjoyed for some time.

---

